# Recounting the history of polyploid research in *D. melanogaster*: 1 century since 2 reports of 3 flies with 4 sets of chromosomes

**DOI:** 10.1080/19336934.2025.2572865

**Published:** 2025-10-10

**Authors:** Lewis I. Held

**Affiliations:** Department of Biological Sciences, Texas Tech University, Lubbock, TX, USA

**Keywords:** *Drosophila*, polyploidy, cancer, neural circuitry, bristle patterns

## Abstract

One hundred years ago, two reports appeared of tetraploid *D. melanogaster* females – curiosities that had never been seen before. The authors, Calvin Bridges and Lilian Morgan, were among the famed founders of fly genetics in T.H. Morgan’s lab at Columbia University. Sadly, their findings have faded into the fog of ancient fly lore. This review exhumes those relics in order to offer modern fly-pushers some possible avenues for polyploid research. That subfield is undergoing a revival that may interest them.

## Introduction

In 1925 Calvin Bridges described two *D. melanogaster* females with twice the normal (diploid) number of chromosomes [1] and alluded to a third such fly that had been found by Lilian Morgan, who was working in the same legendary laboratory of Lilian’s husband, T.H. Morgan. Calvin had recently proposed his ‘balance theory’ [2], wherein a fly’s sex is determined by the ‘X:A’ ratio of the number of X chromosomes to the number of sets of autosomes (A). Ordinary males and females would be 0.5 and 1.0 respectively, with the presence or absence of the Y chromosome playing no role. Bridges had studied various kinds of diploid and triploid flies, all of which matched his model, but the sudden appearance of tetraploids in one of his stocks offered him the chance to test it even further:

The list of sex-types has been enlarged by the discovery of tetraploids, or 4N individuals. These are females, quite identical with normal females in sex characteristics. The tetraploid arose in a stock of triploids; and was detected only by the strikingly different offspring given. … All offspring were triploid females (about 30) or triploid intersexes (about 20). There were no 2N offspring or supersexes. It was seen that this result might be produced if the mother were 4N instead of 3N. For in that case all the reduced eggs would be 2N; and these fertilized by X sperm would give 3N females, and fertilized by Y sperm would give 2X,3A intersexes. … Soon after this first case of 4N female a second similar case was found. Also L.V. Morgan found a third case and was able to prove by genetic tests that four separate X-chromosomes had been present (in press) [1].

Later that same year, Lilian announced her discovery of the third tetraploid (4n) fly that Calvin had mentioned, and she tallied all of the various offspring from this female after she mated her with a wild-type male:

Still another instance of increase in the number of chromosomes was found in a daughter of a 3*n* female, which proved to be a 4*n* female; she behaved genetically as would be expected if she had four X chromosomes and four sets of autosomes. … The 4*n* female was mated to a male which was wild-type in respect to sex-linked characters. Her offspring … were all (with one exception) either 3*n* females or intersexes; none of them was a 2*n* female or a male. The regular eggs of a 4*n* fly would be expected to have two X chromosomes and two sets of autosomes, and these fertilized by sperm with one set of autosomes would give only offspring with three sets of autosomes. The eggs that were fertilized by X sperm should be 3*n* females, those by Y sperm should be intersexes (2XY 3A) [3].

Surprisingly, no further reports of adult flies with a 4n level of ploidy have appeared among the 48 articles that have cited Lilian’s paper in the comprehensive *Web of Science* database in the last 100 years, though 3n strains are commonplace. Why should we care if these 4n studies fade into obscurity? This essay addresses that question. It was written as a centennial tribute and as a review of recent attempts to revive this line of research. Excellent reviews by other authors have extolled the utility of polyploids in general from the standpoint of physiology [4] and cancer [5].

Regrettably, Lilian never enjoyed Calvin’s fame, despite her megawatt brilliance among the godlike geniuses in T.H. Morgan’s lab [6]. All of us working on fly genetics should treasure the legacy of insights that those intrepid pioneers left us [7], including the finding of 4n flies.

## Fankhauser’s law

In the interest of full disclosure, I admit to being bewitched by the phenomenon of polyploidy ever since I read Fankhauser’s seminal review [8] when I was a graduate student 50 years ago. He showed that cell size in salamanders increases with ploidy, while body size remains relatively constant. These correlations, which I refer to as Fankhauser’s Law, appeared to apply to animals more broadly, though nematodes are an exception due to their hard-wired cell lineages [9]. At that time, fruit flies had not been studied anatomically beyond the superficial descriptions that were given in the Bridges and Morgan papers.

A dependence of cell volume on ploidy seemed logical since more chromosomes should yield more proteins and hence fatter cells, but the lack of significant impact of ploidy on body size indicated some sort of constraint. This constraint was easy to explain by Lewis Wolpert’s theory of pattern formation [10], which had become dogma in developmental biology by the mid-1970s. One only needed to envision each body part as specified by morphogen gradients, whose linear dimensions are fixed by the diffusion parameters of the signaling molecules, regardless of the size of the cells within that structure. A leg, for example, should always span the same length, regardless of the sizes of its constituent cells – analogous to a football field whose goal posts are fixed, regardless of whether the players upon it are fat or thin.

**Figure 1.**
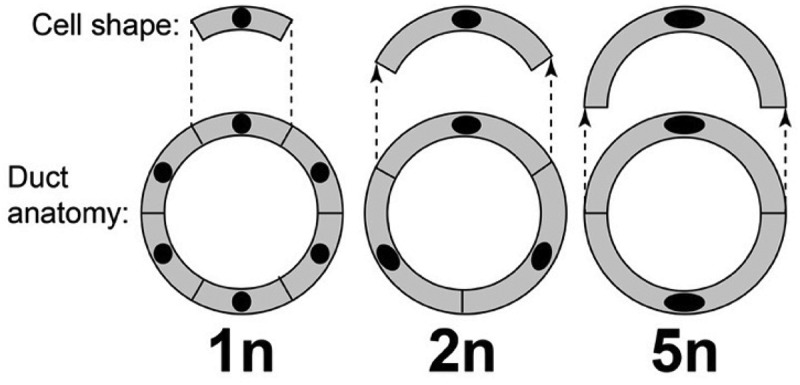
Cell shape (above) within the walls of pronephric ducts (cross-sectioned below) of salamander larvae having ploidies from 1n to 5n. Note the adaptive change in cell shape from a short arc (1n) to a half circle (5n). Adapted from [11].

It stands to reason that a body of fixed size with larger cells must have fewer of them—e.g. 4n individuals would have half as many cells as 2n ones. Might such reductions in cell number affect the anatomy or physiology of an animal’s organs in a measurable way? Fankhauser probed this question in a second paper published later that same year (1945) dealing with the same salamander species [11]. His investigation revealed something striking about the larval pronephric ducts. The diameter of those ducts remains constant with increasing ploidy, as does the thickness of their walls – both traits being essential for duct function. However, those walls must be formed from fewer cells, and to compensate for this reduction the higher-ploidy cells change their shapes (Figure 1).

## Puzzles galore

We do not yet understand how embryonic cells ‘know’ that they should change their geometry in such novel situations. John Gerhart and Marc Kirshner have argued that this flexibility is hard-wired into how organs are built in general [12], and Michael Levin has even gone so far as to attribute this adaptability to a primitive level of consciousness, which he imagines can somehow emerge within cellular aggregates. (Italics are mine.)

Functional anatomy is both robust and plastic, *using basal cognition of cell collectives* to achieve specific anatomical goals. … At the same time, the process reveals important plasticity - the ability to achieve the correct functional anatomy in novel ways that use mechanisms, or traverses configurations, very different from the normal course of events. … The same scheme of exploiting novel mechanisms to achieve the same goal is seen in polyploid newts. In normal animals, small cells use cell-cell coordination mechanisms to arrange into kidney tubules; but when polyploid animals with huge cells are artificially created, individual cells wrap around themselves (a cytoskeletal, unicellular behavior) to create tubules of the same shape and diameter [13].

Regardless of whether assemblies of ordinary cells can ‘think’ in the usual sense of that term, we know that one special category of them can actually do so – namely, the neurons of the animal brain! Polyploids offer a chance to probe the extent to which cognition, memory, and intelligence depend on the number of neurons and their connections. Such questions have often been asked with regard to evolution across the primate clade [14], but we’d like to know what correlations exist *within* a species. Octoploid (8n) mice have been produced [15], but their IQ hasn’t been tested to see if they’re as stupid as we would expect.

The effects of ploidy on kidney cell shape that Fankhauser noticed (Figure 1) would have been subtler – and perhaps even beyond detection – if he hadn’t had a 5-fold range to work with. The lesson for those who would pursue a similar quest in flies is that we should strive for the highest possible ploidy to detect analogous changes: comparing 3n with 2n flies may not suffice. That is why ≥4n flies are desirable, and if we could create ≥4n flies in large quantities, rather than just one or two at a time, then we could probe micro-anatomy with more precision. Aside from the tantalizing mysteries about neural circuitry at the single-cell level [16–18], alluring riddles abound for other tissues in flies that might be approachable using hyperploids.

## Bristle patterns

*D. melanogaster* is covered with bristles that are organized into intricate patterns [19]. How might changes in cell size and/or number affect those patterns? As part of the research that I conducted toward my Ph.D., I decided to explore this riddle [20]. Each leg segment has straight rows of bristles spanning its length, and within each row the bristles are evenly spaced – every 5 cells or so. If the fly were using a cell-counting mechanism to place its bristles (like a farmer planting corn seeds in his field), then increases in cell size should push the bristles apart and reduce their number. In fact, triploid flies do turn out to have larger intervals and fewer bristles, and the interval length is directly proportional to cell diameter [21].

**Figure 2.**
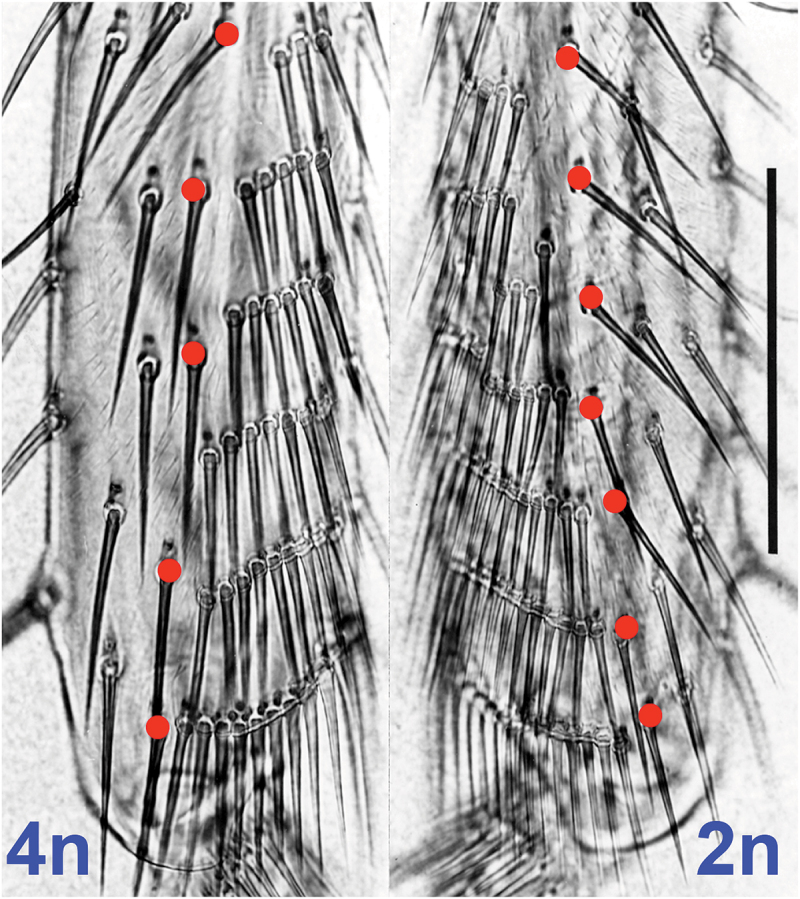
Tetraploid (4n, left) versus diploid (2n, right) tibias of a putative 2n/4n mosaic fly (anterior aspect). Red dots mark bristles in the same longitudinal row. This row borders a triangular chevron of transverse rows, which resembles icicles on windowsills. Intervals are larger on the 4n tibia, and the bristles themselves are bigger. Scale bar = 100 microns. From [23].

This trend also holds for 4n tissue, which I induced in 2n embryos by exposing them to 5000 pounds of hydrostatic pressure per square inch. Such pressure dissolves spindles [22], thereby doubling the ploidy of cells undergoing mitosis at that instant. This treatment only affects a subpopulation of cells, resulting in a 2n/4n mosaic fly that is composed mostly of 2n cells but which exhibits patches of 4n cells engendered by pressure (Figure 2) [23].

## Making polyploids

Aside from pressure, there are various ways to disable spindles and double ploidy. Ben Stormo and Donald Fox were able to create 2n/4n mosaic flies by transiently overexpressing the cyclin-destroying gene *fizzy-related* [24]. Another method that we have used successfully with fly embryos [25] and adult females [26] entails exposure to cold temperatures at or near the freezing point of water [27]. From our treated embryos we were able to obtain 2n/4n adult mosaics, and from our treated mothers we were able to harvest 3n F_1_ offspring, but in neither case did we achieve the production of ≥4n adults.

A more prevalent approach for boosting ploidy relies on the anti-mitotic drug colcemid [28]. We fed fly larvae food containing colcemid in an effort to increase the ploidy of their germ cells from 2n to 4n so as to produce 2n eggs or sperm, which, after fertilization by a 1n wild-type sperm or egg, would yield 3n F_1_ offspring. The drugged larvae had the dominant markers *vestigial*^*Ultra*^ (*vg*^*U*^) or *Glazed* (*Gla*) on their 2^nd^ chromosomes, which allowed 3n F_1_ offspring to be identified by their *vg*^*U*^*/Gla* phenotype as a result of nondisjunction. We obtained 145 3n F_1_ flies [29] but found no evidence of ≥ 1 doubling per larva that would have led to the production of ≥5n F_1_ flies.

In 2023, a new way of generating ≥3n flies was reported by Alexis Sperling et al. in *Current Biology* [30]. The authors used CRISPR to genetically engineer a *D. melanogaster* strain whose virgin females can lay viable eggs without ever mating. How does an unfertilized 1n egg manage to overcome the lethality that prevents 1n embryos from surviving to adulthood [31]? Sperling et al. showed that the mechanism of parthenogenesis here involves the 1n egg nucleus fusing with either 1, 2, or all 3 of its 1n polar body nuclei (its sister and 1^st^ cousins from meiosis) to yield 2n, 3n, or 4n embryos that can apparently develop fully. The authors’ surmisal of 4n survival to adulthood is unproven because they had to kill them as larvae to karyotype their brains. Hopefully, a new protocol for karyotyping mature flies should let us bypass this limitation [32] to verify the 4n status of impaternate F_1_ offspring.

Unfortunately, the fecundity of the virgins in this parthenogenetic stock is so low that < 0.001% of their eggs survive, and less than 10% of those survivors appear to be 4n. We attempted to increase fertility by crossing them with *tudor*-derived spermless males, but failed [33]. We are now trying a different way of boosting fertility by mating the virgins with the male-sterile mutant *ms(3)K81*, which Yoshiaki Fuyama found can prod egg nuclei to fuse with polar bodies at a higher frequency [34]. We are also trying to repeat Fuyama’s investigation of a gynogenetic strain which he showed can produce up to 80 ≥ 3n F_1_ offspring per mother per week [35].

## Number constancy?

Assuming these challenges can be overcome and ≥4n flies become routinely available, one question we are eager to answer concerns stretch-activated sensilla [36]. Humans sense the posture of our arms and legs by proprioceptors in our joints [37], and so do flies [38], but there are key differences. First, a fly’s sense organs are embedded in its exoskeleton and hence easily visible without dissection [38]. Second, those organs consist of only a few cells each and hence should be more sensitive to ploidy effects. And third, they occur in clusters that have a constant number of sensilla [39]. For example, the anterior trochanter has a nest comprised of *exactly* 5 campaniform sensilla (C.S.) on all three legs. How are such fixed numbers specified by the genome? We do not yet know.

The great British polymath John Maynard Smith once wrote an essay entitled ‘The Counting Problem’ [40], where he mused about various ways that cells might ensure precise numbers. Hyperploid flies would allow us to easily disprove some of those strategies. For instance, if the genome dictates 5 C.S. in a trochanter nest, then ≥4n flies should have 5 C.S. there, just like 2n individuals. But if the genome instead allocates a fixed area of cuticle that is normally filled by 5 cells in a 2n fly, then ≥4n flies should have fewer than 5 C.S. in that nest because 5 fatter cells would not be able to fit into that space. This example is just one of many brainteasers that hyperploid flies might help to solve.

## Prospects for brain science

The greatest benefit that ≥4n flies could offer resides in the realm of neuroscience. Doubling the size of neurons would make it easier to insert electrodes for intracellular recordings of action potentials [41], and halving the number of neurons would let us investigate how circuits within the fly connectome [42] adjust to having fewer cells. Some of these goals can be accomplished via currently available 2n/4n somatic mosaics, but the dependence of learning, memory, and intelligence upon brain size can best be addressed when cell number is reduced throughout the entire central nervous system.

## Conclusions

Given the mechanical difficulties that the cells of autopolyploids must face in segregating their chromosomes during mitosis and meiosis [43–46], there would have been good reason – before 1925—to question whether 4n flies could even exist at all, let alone be fertile [47,48].

Autopolyploids face a distinct challenge relative to allopolyploids as they do not have differentiated sub-genomes, and generally lack recombination partner preferences. Somehow these species must sort and recombine four or more highly similar homologous chromosomes during prophase I, and come out the other end (in metaphase I) with a viable array for chromosome segregation [49].

What Calvin Bridges and Lilian Morgan proved 100 years ago is that 4n ‘pink unicorns’ *can* exist in *D. melanogaster* and be fertile. Ironically, a moment’s reflection – literally looking at ourselves in a mirror – should have convinced us that high-level autopolyploids can survive, since we are one! Humans are 8n descendants of 2n chordate ancestors who underwent two doublings of their genomes [50]. Indeed, that’s why we have *four* Hox complexes compared with fruit flies, which have only *one*—albeit one that famously fractured into the separate *Bithorax* and *Antennapedia* clusters of Hox genes long ago [51].

## Data Availability

Data available on request from the author.
